# Remedy for Deafness

**Published:** 1849

**Authors:** Charles Brackett

**Affiliations:** Rochester Indiana


### ARTICLE .II.

*Remedy for Deafness in cases of destruction of the Tympanum and small bones of the ear; with remarks.* By Charles Brackett, M. D. of Rochester Indiana.

In the September No. of the London Lancet, I think, is a very interesting communication on a new method of treating deafness resulting from destruction of the Membrana Tympani—cases generally considered beyond the reach of surgical skill. The treatment is simple, consisting in nothing more than the introduction of a small pellet of fine, clean, moistened cotton, "to a particular place in the bottom of the Meatus Externus." The writer does not seem to know *where* this place is, but says it must be found by manipulating, or moving the cotton well moistened in the bottom of the Meatus, "till the particular spot is reached." After I had read this case I thought that of course the *particular spot* must be in the ulcerated opening in the membrane and probably *in* contact with the malleus, thus restoring a more perfect communication through the Incus and Stapes to the internal ear, where the Portio Mollis the true seat of Audition is distributed.

As there was a patient of mine in town (Rochester) laboring under this disagreeable affliction I determined to test the treatment on him. He is a young man of about twenty years of age, and lost the Tympana of both ears during infancy from ulceration.

I introduced a pellet of cotton wet with water, carefully to the bottom of the Meatus and into the opening in the Tympanum; he then became conscious that he could hear much better than before. I then tried the other ear in the same way. In this ear the passage of the cotton simply *into* the opening of the Tympanum did not relieve the hearing. I then passed it *through* in the *cavity* of the Tympanum when I found that by pushing it lightly up he could immediately hear distinctly as with the other ear. This ear he said "was worse than the other, and discharged a great deal more;" judging from this and that the cotton had to be introduced farther than in the other ear, I concluded that the Malleus at least, if not the Orbiculare and Incus, had been separated and discharged from their places in the cavity. For I consider that the moistened cotton receiving the undulations of sound, and being in contact with the long chain, conveys the vibrations to this chain and through it to the Vestibule where, with the semicircular canals and cochlea, we have the Portio Mollis expanded to receive and transmit the vibrations to the Brain. Such, judging from this case and cases reported in the Lancet, were the impressions I received respecting the modus operandi of the wetted cotton restoring a fine sense of hearing, which lasts so long as the cotton remains moist with the water.

This certainly is but a temporary relief, as the cotton must be renewed as often as it becomes dry, or is displaced from the "*particular spot*" on which it must be to insure success, but I hope the complete temporary relief afforded by the cotton may induce research into the subject which shall result in the discovery of a more permenant means of relief.

